# Effects of rainfall, temperature and photoperiod on the phenology of ephemeral resources for selected bushveld woody plant species in southern Africa

**DOI:** 10.1371/journal.pone.0251421

**Published:** 2021-05-11

**Authors:** Alan Barrett, Leslie Brown

**Affiliations:** Applied Behavioural Ecology and Ecosystem Research Unit, Department of Environmental Sciences, University of South Africa, Florida, South Africa; Chinese Academy of Sciences, CHINA

## Abstract

Variability of ephemeral resources provided by woody plants is related to fluctuating environmental conditions, specifically the predominant climate variables temperature and rainfall. Photoperiod has less impact but also plays a role in the onset of resource pulses. In the seasonally affected bushveld of southern Africa, declining resources could have dire consequences to various animals that depend on these resources. Understanding the impact that rainfall, temperature and photoperiod has on woody plant resources allows managers of natural areas to plan for times when resources are scarce. Using a series of General Linear Models, this baseline study investigates the effects that these variables have on flower production, numbers of new fruit/pods and numbers of new leaves for 113 tagged trees from 26 woody plant species. Leads, lags and coincidental relationships observed between environmental predictor and phenological response variables were explored using time-series cross-correlations and concomitant correlograms. Model results indicated that temperature was the predominant indicator for flowering, with initial flowering starting when temperatures increase in September. A significant lead was observed between flowering and rainfall, suggesting that flower numbers increase approximately one month before rainfall increases. Temperature had the biggest effect on the number of species with new fruits and pods. Significant lags were observed between new fruits and pods and all environmental variables investigated, indicating that these resources depend on rainfall, temperature and photoperiod to reach their full potential. Photoperiod, temperature and the interaction between these variables had a noticeable effect on the number of species with new leaves. Peaks in species with new leaves coincide with peaks in rainfall, temperature and photoperiod. No leading or lagging indicators were observed between new leaves and the environmental variables investigated. In areas containing wildlife populations, recommendations are to undertake regular monitoring of climatic variables investigated, and the ephemeral resources on woody plant species.

## Introduction

Phenological studies investigate the timing of cyclic and seasonal natural phenomena and are essential for determining how plant and animal species respond to changes in environmental variables and, recently to climate change and global warming [1–5]. Current climate change literature predicts changes to global temperatures which will have a direct impact on the intensity and amount of precipitation that different areas experience [6], which in turn will affect the production of resources on woody plant species [2, 7].

Plant phenology, for the purposes of this study, is the investigation of seasonal events resulting in changes to available plant resources including the abundance of flowers, new fruit/pods and new leaves. These plant phenological events are related to various environmental factors including, but not limited to, rainfall, temperature and photoperiod. Understanding phenological events and their drivers provides important information about ecosystem functioning [1, 3, 8].

Changing temperatures and rainfall patterns affect resource availability for woody plant species, influencing the temporal and spatial distribution patterns of animal species that depend on these resources [1, 9, 10]. In temperate areas that have distinct wet and dry seasons, the impact of changing temperatures and rainfall is pronounced [2, 7–11]. Temperature increases and fluctuating rainfall affects plant life cycles in seasonal environments like the southern African bushveld [11–13]. Shifts in plant life cycle events like flowering have disruptive repercussions for animals that depend on particular plant life-cycle phases for their existence [14]. For example, shifting of seasons affect insect pollinators that emerge to take advantage of flowers, and migrating birds that in turn arrive to exploit the insects.

In southern Africa, the majority of natural areas managed for biodiversity, including large nature reserves and national parks, are located in the savanna biome, which is the most widespread biome in Africa [15, 16]. Globally, savanna’s exhibit seasonality, with warm wet summers and cold dry winters [12, 15, 17].

Stocking rates and the variety of animal species that an area can sustainably support are determined using dry season resource phenology estimates [18]. Wildlife conservation managers actively manage the areas that they are responsible for to prevent overstocking and the deleterious impact it could have on the environment. By virtue of their role as conservation managers, monitoring resources is important for determining animal stocking rates to ensure ecosystems remain viable and productive [19–22].

Despite the importance of phenology to biodiversity and wildlife conservation, only one local study that investigated the impact of climate variables on phenology for two woody plant species, over what appears to be a one year period, could be found from literature [23], indicating the general lack of research into this topic in southern Africa. The objectives of this baseline study are to investigate the short-term seasonal effects of rainfall, temperature and photoperiod on the numbers of selected woody plant species flowering, with new fruit/pods and with new leaves at Loskop Dam Nature Reserve, South Africa. We examine data collected over the first five years of an ongoing long-term study initiated in 2009. Our study focuses on 113 selected and tagged bushveld trees from 26 different woody plant species. The study had no deleterious impacts to any plant or animal species.

## Materials and methods

### Study area

**Fig 1:**
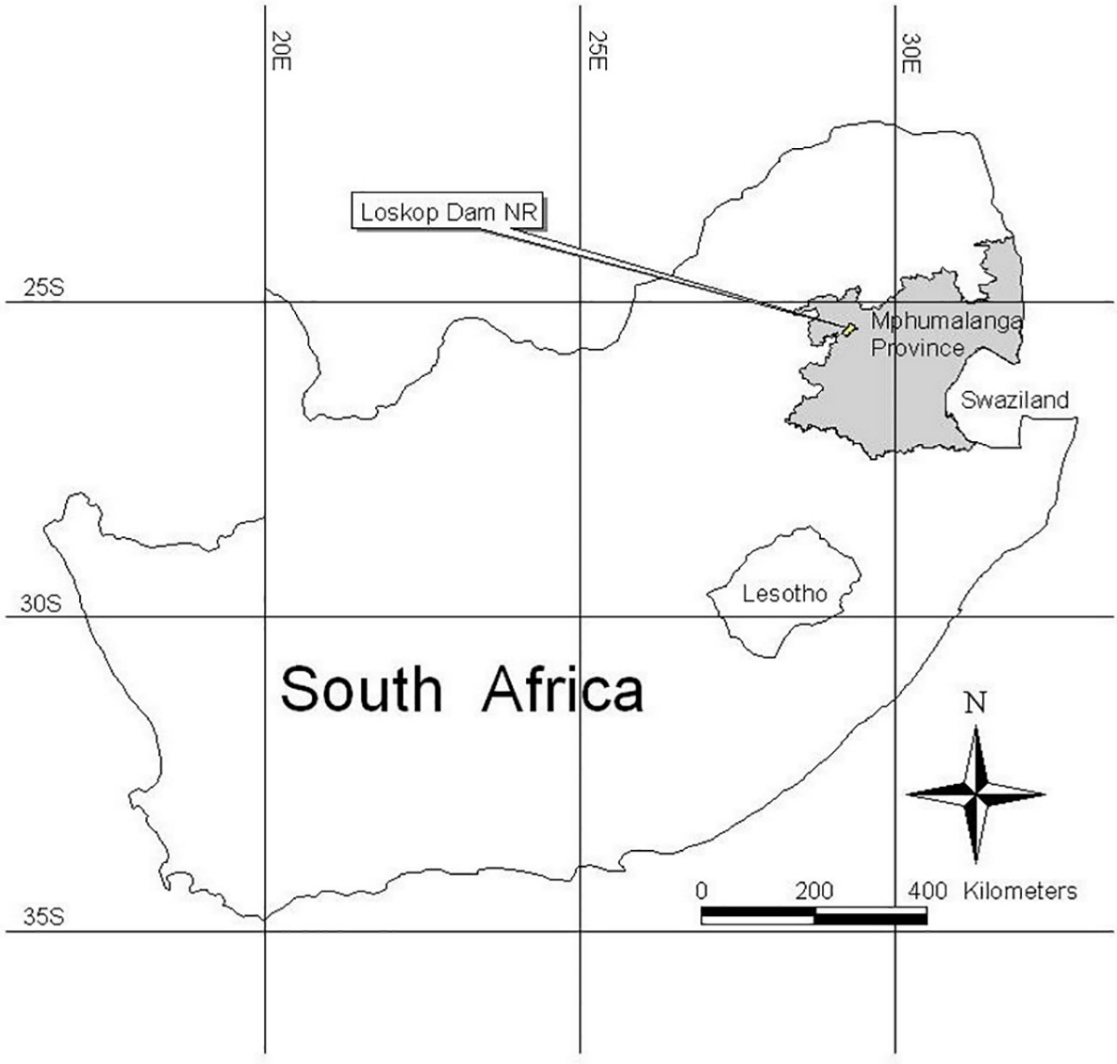
Location of the LDNR within the Mpumalanga province of South Africa [25].

The study site was Loskop Dam Nature Reserve (LDNR), located 52 km north of the town of Middelburg on the N11 national road to the town of Groblersdal in the Mpumalanga province of South Africa (Fig 1). The reserve is located between 29^o^15’00” and 29^o^40’00” East longitude and 25^o^34’00” to 25^o^56’00” South latitude. The reserve is 23 175 ha in size and encompasses the Loskop Irrigation Dam [24].

The reserve’s topography encompasses an incised plateau on higher lying areas comprised of steep cliffs and a variety of slope types, which transition down to the dam through a combination of deep and relatively flat valley bottoms. Altitude ranges from 991 m to 1 420 m above sea level [26]. Overall, the terrain is rugged and broken, attributing towards the heterogeneous nature of the reserve’s vegetation [26].

Loskop Dam Nature Reserve is located in the savanna biome [16]. Savanna is described as herbaceous vegetation with a graminoid lower layer and an upper layer of woody plants that vary from sparse to 75% canopy cover [16]. Vegetation occurring on the incised plateau in the reserve belongs to the Central Sandy Bushveld (SVcb 12) and the Loskop Mountain Bushveld (SVcb 13) Vegetation Units, whereas vegetation occurring in the Olifants River valley belongs to the Loskop Thornveld Vegetation Unit (SVcb 14) [16]. The vegetation of the reserve occurs as a matrix of variation and transitions providing moderately good browsing to the wild ungulates found on the reserve.

Undulating valley bottoms and sandy plains [16] characterize Central Sandy Bushveld. Deep, well-drained soils support tall, deciduous woody plant species including *Terminalia sericea* and *Burkea africana*. Broad-leaved *Combretum* sp. grow on shallow gravely soils. Various *Senegalia* sp., *Vachellia* sp., *Ziziphus* sp. and *Euclea* sp. also occur in this vegetation unit.

Low mountains and ridges containing open tree savanna on lower-lying areas and a denser, mostly broad-leafed tree savanna on lower- and mid-slopes [16] characterize Loskop Mountain Bushveld. The wide diversity of plant communities in the area can be attributed to variations in geology, pedology and topography. Prominent species include *Senegalia caffra* and *Combretum apiculatum*.

Loskop Thornveld vegetation is characterized by valleys and plains of the upper Olifants River catchment [16]. This vegetation unit is comprised of mostly open, deciduous to semi-deciduous, tall, thorny woodlands. Several areas are dominated by *Senegalia* sp. and *Vachellia* sp. that are utilised by browsing ungulates.

LDNR is located in a summer rainfall area with moderate to very hot summers and mild to cold winters. Frost is common on mountain tops and in low-lying valley bottoms [26]. Due to the broken topography of the reserve, there are noticeable local variations in microclimate. Rainfall for Sourish Mixed Bushveld varies from 350 to 650 mm per annum [27]. The majority of rainfall occurs in the summer season (November to April). Temperatures range from a minimum of -8°C to a maximum of 40°C, with an average of 21°C [27].

### Field data collection

Monthly field trips were undertaken to LDNR for plant data collection over a 60-month period from the beginning of May 2009 to the end of April 2014. The current study is part of a long-term phenology project setup to determine the effects of climate variables on resource abundance for various woody plant species utilized by browsing ungulates and primates living on the reserve. During monthly field trips, observations of leafing, budding, flowering and fruiting on 113 tagged woody plants from 26 different woody plant species were undertaken (Table 1).

**Table 1:** Family names, species names and numbers of trees sampled for each species in this study.

*Family*	*Species name*	*Number of trees sampled*
Anacardiaceae	*Lannea discolor*	4
Anacardiaceae	*Sclerocarya birrea*	3
Anacardiaceae	*Searsia leptodictya*	5
Anacardiaceae	*Searsia pyroides*	5
Cannabaceae	*Celtis africana*	5
Combretaceae	*Combretum erythrophyllum*	2
Combretaceae	*Combretum apiculatum*	4
Combretaceae	*Combretum zeyheri*	4
Ebenaceae	*Euclea crispa*	5
>Ebenaceae	*Euclea undulata*	3
Fabaceae	*Dichrostachys cinerea*	7
Fabaceae	*Peltophorum africanum*	5
Fabaceae	*Senegalia burkei*	5
Fabaceae	*Senegalia caffra*	7
Fabaceae	*Vachellia karroo*	5
Fabaceae	*Vachellia nilotica*	6
Fabaceae	*Vachellia robusta*	5
Malvaceae	*Grewia flavescens*	1
Moraceae	*Ficus sur*	2
Olacaceae	*Ximenia caffra*	4
Oleaceae	*Olea europaea s*. *africana*	4
Rhamnaceae	*Berchemia zeyheri*	5
Rhamnaceae	*Ziziphus mucronata*	5
Salicaceae	*Mimusops zeyheri*	5
Sapindaceae	*Pappea capensis*	6
Ulmaceae	*Chaetachme aristata*	1

Prior to the start of the project, monthly reconnaissance trips were undertaken to LDNR for twelve months. The purpose of these trips were to identify and tag woody plant species for phenological monitoring. During reconnaissance trips, all resource bearing woody plant species that provided food items in the study area, and that vervet monkeys (*Chlorocebus pygerythrus)*, baboons (*Papio ursinus)*, giraffe (*Giraffa camelopardalis)*, kudu (*Tragelaphus strepsiceros)*, impala (*Aepyceros melampus)*, bushbuck (*Tragelaphus sylvaticus)* and nyala (*Tragelaphus angasii)* were observed foraging on, were tagged for this study. An attempt was made to tag equal amounts of the different woody plant species, but the number of these species present, and trees that died during the study period, influenced final numbers. Locations of the woody plants were recorded using a Garmin 12XL^TM^ GPS and each plant was numbered with a metal tag that was placed on the southern side of the tree’s trunk at breast height.

On commencement of data collection for the long-term project that is still underway, the following data were collected on a monthly basis for each tagged woody plant: Tag Id; Species Name; Resource Items Present (flowers, new fruit/pods and new leaves); and the visually estimated Relative Abundance of Resources Items available in the tree canopy (0–5%, >5–12%, >12–25%, >25–50%, >50–75%, and >75–100%) for three height classes (0–1 m, >1–3 m, and >3 m). The number of flowers, new fruit/pods and new leaves in a marked 1m^3^ segment of the tree were also counted for extrapolation into the total tree volume [25]. Leaves were classified as leaf buds, new leaves or old leaves; and fruit/pods were classified as new or mature. For this study, we focused on numbers of flowers, new fruit/pods and new leaves because the relationship between temperature, rainfall and photoperiod affects these resource items directly [28].

Rainfall and temperature data for the study period were sourced from reserve records. Climate information is collected daily by reserve staff from a single weather station placed at the reserve’s administration buildings and from rain gauges placed at the various ranger pickets distributed throughout the reserve. Monthly means were calculated using daily data captured for the study period. Daily photoperiod (daylight hours) data were downloaded for Nelspruit from the internet [29] and mean monthly day lengths were calculated for the study period.

### Statistical analyses

Mean monthly values for all data collected during the period were analysed using a series of Poisson family General Linear Models [30, 31]. General Linear Models investigated which of the various sampled environmental variables (rainfall, temperature or photoperiod and interactions between these variables) had the greatest effect on the numbers of woody plant species flowering, with new fruit/pods and with new leaves.

Lags, leads and coincidental relationships between environmental variables and phenological variables were explored using time-series cross-correlations [31] to determine whether numerical responses in phenological variables were associated with the different environmental variables. The statistical package R [32] was used for all statistical analyses.

## Results

**Fig 2:**
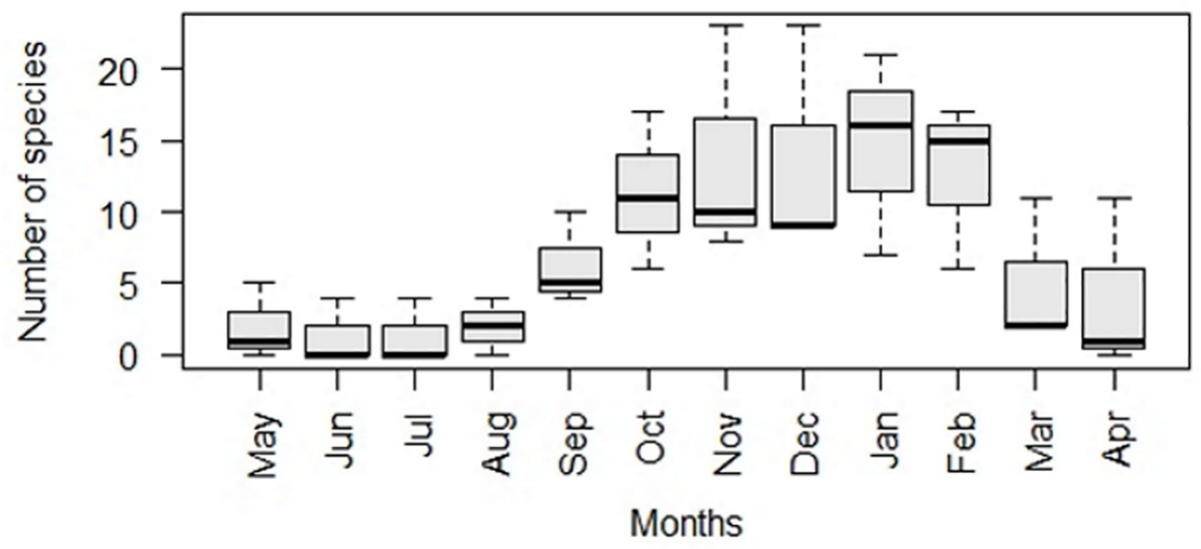
Numbers of woody plant species containing resources (flowers, new fruit/pods and new leaves) for the study period.

Numbers of woody plant species bearing resources (flowers, new fruit/pods and new leaves) began to increase during August and continued to do so until January, as depicted in the box and whisker plots for Fig 2. In January, the maximum number of woody plant species investigated had resources on them. From February to July, the numbers of woody plant species containing resources declined as the autumn period progressed into winter.

**Fig 3:**
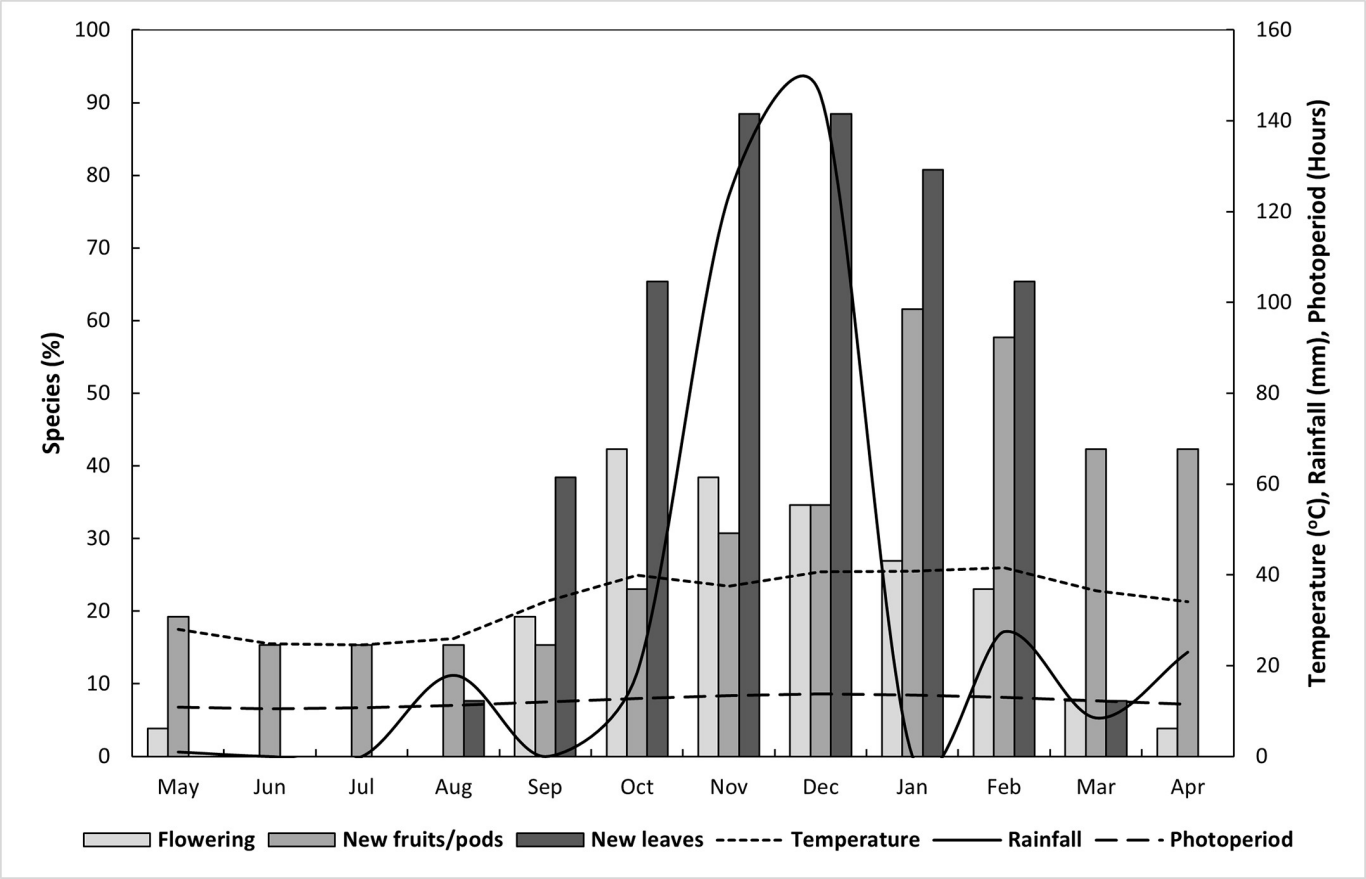
Effects of rainfall, temperature and photoperiod on percentage of woody plant species flowering, with new fruit/pods and with new leaves.

Associations between mean monthly rainfall, mean monthly temperature and mean monthly photoperiod with the percentage of woody plant species flowering, that had new fruit/pods and that had new leaves are depicted in Fig 3.

Initial flowering on the sampled woody plant species started when temperatures began to increase in September and peaked a month later in October (Fig 3 –flowering component). From November to May the number of woody plant species that were flowering declined and between June and August no flowers were present on any of the sampled woody plant species.

After initial flowering started in September, the onset of new fruit/pods became visible a month later in October (Fig 3 –new fruits/pods component). Numbers of woody plant species containing new fruit/pods increased and peaked in January, declining from February to September.

Leafing was independent of flowering and fruiting. Sampled woody plant species started leafing in August and peaked in November and December (Fig 3 –new leaves component). Leafing continued at a reduced rate until March, after which no new leaves were present until August.

A series of General Liner Models were run to investigate the effects of rainfall, temperature and photoperiod on the number of woody plant species flowering, with new fruit/pods and with new leaves. Results for the model with the best fit are presented in Table 2.

**Table 2:** Results for the minimum adequate models for a series of general linear models run to determine the effects of rainfall, temperature and photoperiod on the number of woody plant species flowering, with new fruit/pods and with new leaves.

*Coefficients*	*B*	*SE B*	*β*	*CI*	*P*	
**Intercept: Number of species flowering**	-0.09	5.03	N/A	(-10.11, 9.80)	0.99	
Rainfall	-0.39	0.27	-4.65	(-0.96, 0.10)	0.15	
Temperature	0.76	0.32	0.74	(0.18, 1.46)	0.02	*
Photoperiod	-1.24	0.92	-0.35	(-3.12, 0.48)	0.17	
Rainfall:Temperature	-0.02	0.01	-0.19	(-0.03, 0.00)	0.05	*
Rainfall:Photoperiod	0.06	0.03	0.06	(0.00, 0.13)	0.09	
**Intercept: Number of species with new fruit/pods**	-0.44	0.69	N/A	(-1.86, 0.86)	0.52	
Rainfall	0.00	0.00	-0.02	(-0.01, 0.00)	0.37	
Temperature	0.12	0.03	0.11	(0.06, 0.18)	<0.00	*
**Intercept: Number of species with new leaves**	-129.30	58.28	N/A	(-277.60, -28.49)	0.03	*
Rainfall	-1.76	1.34	-8.90	(-4.50, 0.93)	0.19	
Temperature	3.73	1.80	1.53	(0.73, 8.72)	0.04	*
Photoperiod	12.51	5.73	1.49	(2.35, 26.36)	0.03	*
Rainfall:Temperature	0.12	0.07	0.61	(-0.02, 0.27)	0.10	
Rainfall:Photoperiod	0.09	0.09	0.04	(-0.11, 0.28)	0.35	
Temperature:Photoperiod	-0.39	0.19	-0.05	(-0.86, -0.06)	0.04	*
Rainfall:Temperature:Photoperiod	-0.01	0.00	-0.04	(-0.02, 0.00)	0.13	

B = Beta value, SEB = Standard Error of Beta, β = Standardized Beta, CI = Confidence Intervals, P = Significance

* = Significant result

For the ‘**Intercept: Number of species flowering**’ in Table 2, temperature was the predominant indicator for flowering on woody plant species (*P* = 0.02), followed by the interaction between rainfall and temperature (*P* = 0.05). The negative rainfall-temperature interaction coefficient indicated that the effect of the combined action of rainfall and temperature was less than the sum of their individual effects. For this interaction, the association between rainfall and the number of species flowering decreased if temperature increased. Results for the ‘**Intercept: Number of species with new fruits/pods**’ indicated that temperature had the most noticeable effect on the number of species with new fruit/pods (P < 0.00). Findings for the ‘**Intercept: Number of species with new leaves**’ showed that photoperiod (*P* = 0.03) had the most noticeable effect on the number of woody plant species with new leaves, followed by temperature (*P* = 0.04), and the interaction between temperature and photoperiod (*P* = 0.04). Although both the component predictor variables temperature and photoperiod had positive coefficients, the temperature-photoperiod interaction was negative and the effect of the combined action of the predictor variables was less than the sum of their individual effects. As the value of one of the predictor variables increased, the other decreased and *vice versa*. The positive relationships between temperature and photoperiod were weaker when the values of one of these variables was high. If temperatures were high and day length (photoperiod) was short, or day length was long and temperatures were low, then the number of species with new leaves decreased.

**Fig 4:**
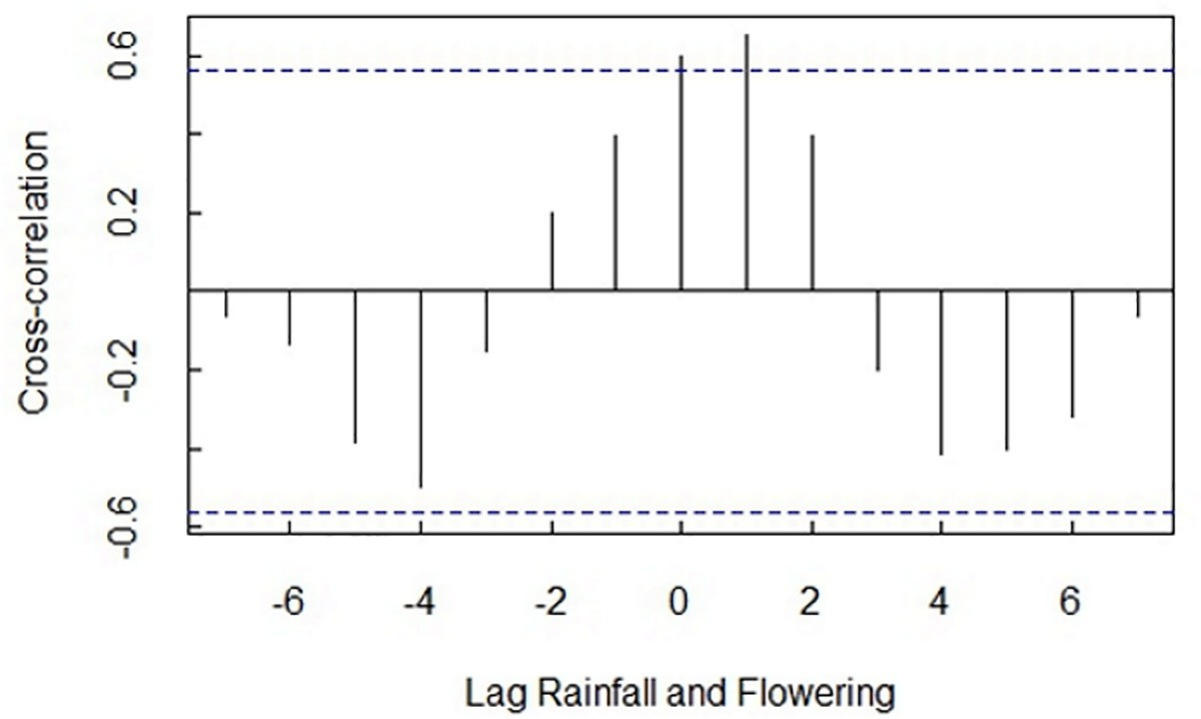
Correlogram indicating a prominent, moderately significant lead between flowering and rainfall at position 1.

**Fig 5:**
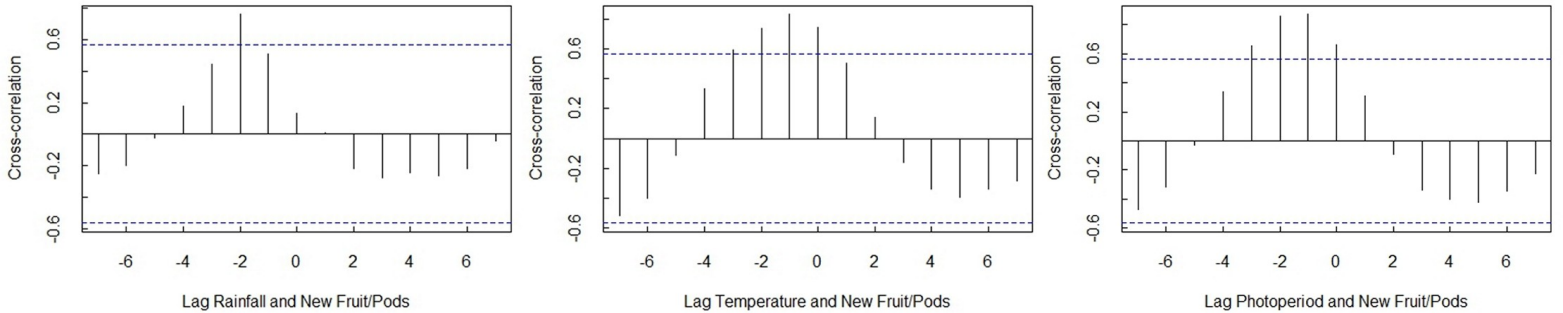
Correlogram indicating moderately significant lags between a) new fruit/pods and rainfall at position -2, b) new fruit/pods and temperature at position -1, and c) new fruit/pods and photoperiod at positions -2 and -1.

Potential lags, leads and coincidental relationships between flowering and the different environmental variables investigated were explored using cross-correlations (Table 3). Lags are represented by negative position values and leads by positive position numbers in the table. Position refers to the position of the lag or lead in associated Figs 4 and 5 and represents the lag or lead in months between the phenological event and the environmental variable correlated to it. Period in Table 3 refers to the number of months between the start and end of a particular phenological cycle.

**Table 3:** Lag and lead results for flowering, new fruit/pods and new leaves with rainfall, temperature and photoperiod. If a lag or lead was present, it is indicated in the ‘position’ column. Noticeable lags and leads were found for flowering and rainfall, and for new fruit/pods with rainfall, temperature and photoperiod. No lags were found for new leaves.

Phenological event	Rainfall						Temperature						Photoperiod					
	Lag/Lead						Lag/Lead						Lag/Lead					
	Position	Period	*r*	*DF*	*SE*	*P*	Position	Period	*r*	*DF*	*SE*	*P*	Position	Period	*r*	*DF*	*SE*	*P*
Flowering	1	10 months	0.65	10	0.24	<0.00	0	12 months	0.82	10	0.18	<0.00	0	12 months	0.88	10	0.15	<0.00
New fruit/prods	-2	12 months	0.76	10	0.20	<0.00	-1	15 months	0.83	10	0.17	<0.00	-2	14 months	0.86	10	0.16	<0.00
-	-	-	-	-	-	-	-	-	-	-	-	-	-1	14 months	0.87	10	0.15	<0.00
New leaves	0	10 months	0.65	10	0.24	<0.00	0	13 months	0.81	10	0.19	<0.00	0	12 months	0.94	10	0.11	<0.00

r = correlation coefficient, DF = Degrees of Freedom, SE = Standard Error, P = significance

A noticeable, moderately significant lead (cross-correlation: *r*(10) = 0.65, *SE* = 0.24, *P* < 0.00) was found between flowering and rainfall at position 1 in Table 3, suggesting that flowers increased approximately one month before rainfall increased. This lead was also evident in Fig 4 at position 1.

As temperatures began to increase in September, the woody plant species investigated started flowering (Fig 3). In addition to the initiation of flowering, temperature increases also influence the development of new fruit/pods (Fig 3). Moderately significant lags were observed between new fruit/pods and all environmental variables investigated (rainfall, temperature and photoperiod). A predominant lag between new fruit/pods and rainfall occurred at lag position -2 (cross-correlation: *r*(10) = 0.764, *SE* = 0.204, *P* < 0.00), indicating that approximately two months after good rainfall there was an increase in the number of plants that had new fruit/pods (Fig 5A). The main lag between new fruit/pods and temperature occurred at lag position -1 (cross-correlation: *r*(10) = 0.834, *SE* = 0.174, *P* < 0.00), indicating that approximately one month after maximum temperatures were recorded there was a noticeable increase in the number of plants that had new fruit/pods (Fig 5B). The predominant lag between new fruit/pods and photoperiod was also moderately significant at lags -2 (cross-correlation: *r*(10) = 0.861, *SE* = 0.161, *P* < 0.00) and -1 (cross-correlation: *r*(10) = 0.873, *SE* = 0.154, *P* < 0.00), indicating that the number of plants that had new fruits/pods increased one to two months following maximum day length recordings (Fig 5C).

As was the case for new fruits/pods, when temperatures began to increase, the sampled woody plant species started producing new leaves and reached peak production when rainfall was highest, declining thereafter (Fig 3). No noticeable lags, leads or coincidental relationships were observed between the various environmental variables investigated and the number of new leaves on woody plant species at LDNR (Table 2).

## Discussion

Over the duration of the study period, various woody plant species at LDNR produced resources intermittently, sustaining the various animal species that depend on these resources. Resources in the form of flowers, new fruit/pods and new leaves were available on different woody plant species throughout the year [25]. Numbers of woody plant species containing resources increased from August/September and peaked in January, declining after this peak until June/July when numbers of trees with resources were at their lowest (Fig 2). Months where the numbers of woody plant species contained the most resources coincided with increased temperatures and rainfall that occurs during the warm wet months.

Predominant indicators for flowering at the study site were temperature followed by the interaction between rainfall and temperature. Initial flowering started in September when temperatures began to increase and peaked in October, declining from November to June when no flowers were present on any of the sampled woody plant species. These results are similar to findings by [33] in China, who found that temperature was the main driving agent for flowering in a common tree species (*Syringa oblata*) monitored for 40 years at 44 phenological observational sites. Researchers [4] also found that temperature variability and lag effects alter flowering trends in subarctic plant communities. In our study, after initial flowering started in September, model results indicated that rainfall in spring was important to sustain flowering. The significant lead observed between flowering and rainfall (Table 3 and Fig 4) suggests that if rainfall is reduced or absent, that flowering will still take place, but fruiting could be limited or absent.

The model with the best fit for new fruit/pods and associated environmental variables indicated that temperature had the most noticeable effect on the number of species with new fruit/pods at the study site. A proportion of the woody plant species present at LDNR had new fruit/pods in all months of the year (Fig 3 –new fruits/pods component). The onset of new fruit/pods began in October and peaked in January, declining from February to September. Since new fruit/pods are the product of flowering, there is an inverse relationship between these phenological events. As flower numbers begin to decline on woody plant species, the flowers become fruit/pods and the numbers of these begin to increase. Significant lags were observed between new fruit/pods and all environmental variables investigated, indicating that new fruit/pods depend on rainfall, temperature and photoperiod to reach their full potential. These lags could, in part, be attributed to the often difficult to observe transition from flowers to fruit production [34], which was not investigated in this study. Even though temperature had the most noticeable effect on the number of woody plant species with new fruit/pods (Table 2), explored lags indicated that rainfall and photoperiod also contributed towards woody plant species reaching their full productive potential (Fig 5).

Photoperiod, followed by temperature, and the interaction between temperature and photoperiod were the main indicators for new leaves on the sampled woody plant species at LDNR. Leafing started in August and peaked in November and December, continuing until March, with no new leaves present between April and July. Peaks in the number of new leaves appear to be coincidental (in synch) with peaks in rainfall, temperature and photoperiod. No leading or lagging indicators were observed between numbers of woody plant species with new leaves and the environmental variables investigated. Since no dependencies were observed between these phenological events, results suggest that the appearance of new leaves is independent of flowering and new fruit/pods.

To summarize, our results indicate that when temperatures begin to increase, flowering begins and new leaves start to appear. After the onset of flowering and leaf budding, rain is required to ensure that these events are sustained and that they reach their full potential [35]. Although no regional studies could be found that investigated the effects of rainfall, temperature and photoperiod on flowering, fruiting and leafing of trees, several studies have investigated leaf phenology for individual tree species [36–38]. Researchers [37] found that temperature was positively correlated with leaf phenology in riverine thickets in the central Free State, South Africa, which is similar to what we found. Our findings are also similar to a study that investigated woody tree flowering and fruiting phenology in a tropical-seasonal rainforest in southwestern China [39]. If rain is delayed or absent, both flowering and new leaf production will be compromised in terms of yields produced [40]. In addition to rainfall, temperature also has a noticeable effect on the number of species with new fruit/pods [2, 4, 33].

Phenological synchronicity in seasonal environments is influenced by the timing and duration of climatic events such as rainfall, changing temperatures and shifts in photoperiod [2]. Climatic variability affects woody plant resource emergence and availability, which in turn has consequences for many animals living in temperate environments that depend on these resources [37, 41, 42]. Temporal shifts in climatic events result in lag effects that produce varying floral syndromes [33]. Prominent lags that were explored in this study showed a recurring tendency of between 10 and 15 months (Table 3). The consequence of asynchronous flowering is asynchronous fruit and pod production.

## Conclusions

The objectives of this study were to investigate the short-term seasonal effects of rainfall, temperature and photoperiod on flowering, numbers of new fruit/pods and numbers of new leaves for selected woody plant species at LDNR. To date, no studies have investigated the relationship between rainfall, temperature and photoperiod on resource phenology for the sampled woody plant species in southern Africa.

Results indicate that there are intricate relationships between the different environmental variables investigated and the phenological events examined. Temperature plays a predominant role in all of the phenological events that were investigated. After winter, in August and September, as temperatures increase, flower and new leaf production starts. For sustained flowering and leaf budding, and for these events to reach their full potential, adequate rainfall is required. If rain is absent or delayed, these phenological events result in the decreased production of flowers and resultant fruits/pods. We also found that temperature has a noticeable effect on the number of species that contain new fruit/pods. These findings indicate the importance of both temperature and rainfall to resource production in the study area and the southern African bushveld in general.

The combination of lags, leads and coincidental relationships found between some of the environmental variables and certain phenological events further highlight the complicated nature of the relationships between these variables and events. Exploration of these lags, leads and coincidental relationships indicate that flowering, new fruit/pod development, new leaf development, and the environmental variables explored are cyclic [43]. Should a major shift in environmental variables take place, as is predicted for climate change, this would have conspicuous effects on the abundance and availability of woody plant resources [2, 4, 7, 44, 45]. A change to ephemeral woody plant resources will have a causal effect on the various animals that depend on these resources [45, 46].

With global concerns about the consequences of changing climate variables to natural environments and ecosystems, it is important to monitor local impacts of changing climate variables, particularly temperature and rainfall [2, 7, 10]. Wildlife managers are in a favourable position to make decisions about grazer and browser stocking rates when they are aware of potential resource problems. Recommendations for managers of natural areas containing animals that depend on ephemeral woody plant resources are that they monitor the effects that rainfall, temperature and photoperiod have on woody plant resource phenology. If resources become less due to shifts in climatic variables, managers need to adjust animal population sizes or consider supplementary feeding to prevent permanent damage to the environment [37]. It is further recommended that future research investigate the impacts of various plant stressors on resource availability and abundance. The survival strategies of various woody plant species should also be investigated to determine how current and future global warming trends impact on these strategies.

## Supporting information

S1 Data (XLSX)
